# HPV E6 and E7 oncoproteins cooperatively alter the expression of Disc Large 1 polarity protein in epithelial cells

**DOI:** 10.1186/s12885-020-06778-5

**Published:** 2020-04-07

**Authors:** María Paula Dizanzo, Federico Marziali, Clarisse Brunet Avalos, Marina Bugnon Valdano, Santiago Leiva, Ana Laura Cavatorta, Daniela Gardiol

**Affiliations:** Instituto de Biología Molecular y Celular de Rosario-CONICET, Facultad de Ciencias Bioquímicas y Farmacéuticas, Universidad Nacional de Rosario, Suipacha 531, 2000 Rosario, Argentina

**Keywords:** DLG1, HPV, HPV oncoprotein, Expression, Cancer

## Abstract

**Background:**

Persistent infection with high-risk Human Papillomavirus (HPVs) is associated with the development of cervical cancer. The transforming capacity of these viruses relies on the cooperative action of the E6 and E7 viral oncoproteins. Among the oncogenic activities of E6, the interaction and interference with cell polarity PDZ proteins have been well established. One of the most characterized PDZ targets of HPV E6 is human Disc large 1 (DLG1), a scaffolding protein involved in the control of cell polarity and proliferation. Interestingly, in cervical squamous intraepithelial lesions, alterations in DLG1 expression were observed in association to tumour progression. Moreover, the expression of both HPV E6 and E7 proteins may be responsible for the changes in DLG1 abundance and cell localization observed in the HPV-associated lesions.

**Methods:**

Due to the relevance of DLG1 deregulation in tumour development, we have performed an in-depth investigation of the expression of DLG1 in the presence of the HPV oncoproteins in epithelial cultured cells. The effects of HPV E6 and E7 proteins on DLG1 abundance and subcellular localization were assessed by western blot and confocal fluorescence microscopy, respectively.

**Results:**

We demonstrated that the relative abundance of HPV-18 E6 and DLG1 is a key factor that contributes to defining the expression abundance of both proteins. We also show here that a high expression level of DLG1 may negatively affect HPV-18 E6 nuclear expression. Moreover, the co-expression of HPV-18 E6 and E7 produces a striking effect on DLG1 subcellular localization and a co-distribution in the cytoplasmic region. Interestingly, HPV-18 E7 is also able to increase DLG1 levels, likely by rescuing it from the E6-mediated proteasomal degradation.

**Conclusions:**

In general, the data suggest that HPV-18 E6 and E7 may have opposing activities in regards to the regulation of DLG1 levels and may cooperatively contribute to its subcellular redistribution in the HPV context. These findings constitute a step forward in understanding the differential expression of DLG1 during tumour progression in an HPV-associated model.

## Background

Persistent infection with high-risk Human Papillomavirus (HPVs), such as HPV-16 and HPV-18, is closely associated with the development of cervical cancer, a highly relevant global health concern [1]. This tumour is the third largest cause of cancer-related deaths in women worldwide, with a notable incidence difference between high and middle/low country incomes (http://globocan.iarc.fr/old/FactSheets /cancers/cervix-new.asp). The transforming activity of high risk HPVs relies on the cooperative action of the E6 and E7 oncoproteins [2]. These viral proteins act in concert to alter the function of key cellular proteins that regulate important oncosuppressor processes [2]. Among the oncogenic activities of E6, the interference with proteins that regulate cell polarity and maintain cell junction integrity has been established as a crucial factor for the HPV-induced malignant transformation. These polarity regulators are characterized by the presence of PDZ (PSD-95/DLG/ZO-1) interaction domains, which can be targeted by a conserved PDZ binding motif (PBM, X-[T/S]-X-V) located at the C-terminal region of E6 (for a review see [3]).

One of the best-characterized PDZ targets of HPV E6 is DLG1, a member of the Scrib polarity complex [4, 5]. DLG1 is a scaffolding protein that coordinates the assembly of multiprotein complexes involved in the control of cell polarity, cell division, cell migration, and intracellular trafficking [6–9]. In epithelial cells, DLG1 co-localizes with E-cadherin at the adherens junctions in association with the cytoskeleton, where it has both structural and signalling functions [9, 10]. Initial evidence indicating roles for DLG1 in cell growth regulation come from experiments in *Drosophila*, which demonstrated that the loss of DLG1 expression leads to uncontrolled epithelial cell proliferation and neoplastic transformation, suggesting DLG1 as a tumour suppressor. This concept was supported by the identification of its ability to interact with several regulators of cell proliferation, such as Adenomatous polyposis coli and phosphatidylinositol-3,4,5-trisphosphate 3-phosphatase, and by the recognition of its capacity to arrest cell cycle progression in cultured cells when is overexpressed [11, 12]. Furthermore, DLG1 expression was shown to be low or even absent in the latest stages of tumour development in different cancers, which strongly suggest oncosuppressor functions [13].

The discovery of DLG1 targeting by different viral oncoproteins, including HPV E6, has emphasized the role of DLG1 in tumour development. Initial studies indicated that an overexpression of E6 could stimulate DLG1 degradation through the proteasome-dependent mechanism [5]. Interestingly, this ability is not conserved in low-risk HPV E6 proteins (not associated to cancer development), which are unable to interact with DLG1 due to the lack of the C-terminal PBM. These facts support a key role for DLG1 targeting in HPV-induced cell transformation [5, 14]. These observations encouraged us, and others to analyse in detail the expression of DLG1 in cervical lesions by immunohistochemistry (IHC). In invasive cervical carcinomas, the abundance of DLG1 was very low or almost undetectable. However, in some squamous intraepithelial precursor lesions (SIL), DLG1 levels were increased in comparison to the normal epithelium, and its cellular localization was altered, being redistributed from the cell contacts to the cytoplasm [15, 16]. Interestingly, such differential expression was demonstrated to be a very efficient marker of progression of low grade SILs (LSILs) to malignancy. Indeed, LSILs showing an accumulation of DLG1 in the cytoplasm progressed to high-grade SIL (HSIL), whereas those that retained DLG1 expression at the cell borders regressed to normal epithelial [17]. The observed changes in DLG1 expression could be significant for HPV pathogenesis since DLG1 normal functions strictly rely on its correct subcellular distribution [9, 10].

Notably, in organotypic epithelial raft cultures generated from human keratinocytes expressing HPV-18 E6 and E7 oncoproteins, we detected an increased and mislocalized expression of DLG1, in agreement with the observations in SILs biopsies [18]. This finding may indicate the participation of these viral proteins in DLG1 deregulation, and ultimately during this pathological process.

Thus, the current evidence indicates that the relationship between HPV and polarity proteins could be more complex than previously thought. The E6-DLG1 interaction may not always promote DLG1 degradation, but it could play a different role during the oncogenic development. In line with this, it is possible that such interaction not only abolishes DLG1 tumour suppression activities, but also stimulates potential oncogenic functions of DLG1, enhancing both viral replication and transformation. In agreement with this, the interaction of the Human adenovirus E4-ORF1 oncoprotein with DLG1, in a PDZ-dependent manner, promotes the phosphatidylinositol-4,5-bisphosphate 3-kinase signalling activation, suggesting unexplored oncogenic traits for DLG1 [14]. Furthermore, the E6-DLG1 association may be required in different stages of the viral cycle. Concurring with this, it has been reported the importance of the E6 PBM, and the interaction with PDZ proteins for a productive HPV replicative cycle. Indeed, the E6 PBM was shown to participate in the survival and expansion of HPV-infected keratinocytes, thereby favouring genome amplification [19–21].

Interestingly, our previous studies also suggest a role for HPV E7 in the differential expression of DLG1, as the single presence of this viral protein promotes an increase in DLG1 abundance in raft tissues and in monolayer epithelial cell cultures [18]. HPV E7 deregulates signal transduction pathways involved in cell proliferation and transcriptional regulation in order to favour viral replication. Moreover, it induces changes in the expression of cell junction proteins, like E-cadherin at the adherens junctions [22, 23]. Apart from that, E7 proteins derived from cutaneous HPVs increase the abundance of epithelial anchor proteins, such as β-catenin and zona occludens-1 [24]. These findings altogether suggest still underexplored activities of HPV E7 associated to alterations in cell-to-cell contacts and polarity that deserve further investigation.

Due to the relevance of DLG1 deregulation in tumours, we have performed an in-depth investigation of the expression of DLG1 in the presence of the HPV oncoproteins in epithelial cells. We have demonstrated that the relative abundance of HPV-18 E6 and DLG1 is a key factor that contributes to defining the expression levels of both proteins, suggesting different outcomes for the HPV-18 E6-DLG1 interaction depending on the biological context. We also show here that a high expression level of DLG1 may negatively affect HPV-18 E6 nuclear expression. Moreover, the co-expression of HPV-18 E6 and E7 proteins produces a striking effect on DLG1 subcellular localization and a co-distribution at the cytoplasmic region. Furthermore, HPV-18 E7 is able to increase DLG1 levels, likely rescuing it from the E6-mediated degradation. These data provide new insights for a more complete understanding of the differential expression of DLG1 during tumour progression in an HPV-associated model.

## Methods

### Cell culture and transfection

Human Embryonic Kidney 293 epithelial cells (HEK293, ATCC #CRL-1573) and A549 lung epithelial cells (ATCC CCL-185) were grown in Dulbecco’s modified Eagle’s medium DMEM (Gibco, NY, USA) supplemented with 10% (v/v) fetal bovine serum (Natocor, Córdoba, Argentina). The cultures were maintained at 37 °C, in a 5% CO_2_ atmosphere. All cell lines were regularly tested for mycoplasma contamination using the mycoplasma contamination Corning kit. For cell transfection, the calcium phosphate method was used as previously described [25].

### Plasmids

The coding sequence of HPV-18 E6 (E618) oncoprotein was cloned into the pseyfp2-c1 (expression plasmid encoding of the super enhanced yellow fluorescent protein 2) and pmTurq2-c1 (encoding an enhanced version of the cyan fluorescent protein) vectors to obtain pseyfp2-E618 and pmTurq2-E618. pseyfp2-E618 construction was used as template for obtaining the seyfp2-E618ΔPBM mutant version in which the E6 PBM (E T Q V, C-terminal aminoacids) was deleted by PCR mutagenesis. The HPV-18 E7 (E718) coding sequence was amplified by PCR from pcDNA3-His-E718 [18] and cloned into the pLPC-mCherry and pegfp-c1 vector, resulting in pLPC-mCherry-E718 and pegfp-E718. The mTurq2-DLG1 and pcDNA3-HA-DLG1 (DLG1 protein fused to the HA, human influenza hemagglutinin epitope) vectors were previously described [5, 26]. A plasmid encoding β-galactoside (β-Gal) protein was included in all transfections as a control of equal transfection efficiency.

### Antibodies

Antibodies used were mouse monoclonal anti-HA (12CA5, Sigma Aldrich, St. Louis, MO, USA), rabbit monoclonal anti-green fluorescent protein (GFP) (FL, Santa Cruz Biotechnology, Santa Cruz, CA, USA), mouse monoclonal anti-DLG1 (2D11, Santa Cruz Biotechnology, Santa Cruz, CA, USA), mouse monoclonal anti- γ tubulin (GTU-88 Sigma Aldrich, St. Louis, MO, USA) and mouse monoclonal anti- β-Gal (Promega, Madison, USA).

### Cell protein extracts and Western blot

Whole-cell extracts were prepared by resuspending cells directly in SDS-sample buffer (125 mM Tris–HCl pH 6.8, 2% SDS, 20% glycerol, 0.01% bromophenol blue and 10% b-mercaptoethanol). For obtaining soluble and insoluble protein pools cells were harvested in E1A lysis buffer (50 mM 4-(2-hydroxyethyl)- 1-piperazineethanesulfonic acid (HEPES), 250 mM NaCl, 1 mM MgCl_2_, 0.1% NP-40) containing 1% Halt Protease inhibitor cocktail (Thermo Fisher Scientific, Rockford, IL, USA). Such protein extracts were centrifuged at maximum speed, and the supernatant was separated and considered to be the detergent soluble fraction, whereas the remaining pellet was considered to be the insoluble fraction. When specified, cells were treated with the proteasome inhibitor bortezomib (BTZ; Santa Cruz Biotechnology, Santa Cruz, CA, USA) 14 μM for 4 h prior to protein extraction.

**Fig. 1:**
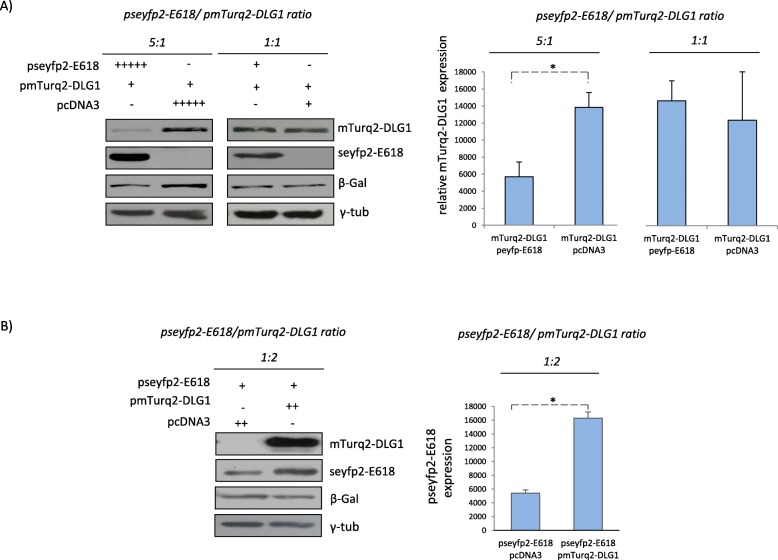
DLG1 and E618 protein levels are dependent on their relative abundance. **a** DLG1 levels decrease when the E618 oncoprotein is expressed at high levels. Different pseyfp2-E618/pmTurq2-DLG1 plasmid DNA ratios were transfected into HEK293 epithelial cells (5:1 and 1:1 μg). pcDNA3 was used as empty vector control. Middle and Right panels, densitometry analysis of western blots for DLG1 expression for equal γ tubulin levels in the different E6:DLG1 ratio conditions (mean ± SD, *n* = 3). Asterisks denote significant difference tested by one-tailed t-test (*p < 0.05). **b** High levels of DLG1 promote an increment in E618 levels. A 1:2 μg ratio of pseyfp2-E618/pmTurq2-DLG1 plasmid was used. pcDNA3 was used as empty vector control. Right panel, densitometry analysis of western blots for E618 expression for equal γ tubulin levels (mean ± SD, *n* = 3). Asterisks denote significant difference among conditions tested by one-tailed t-test (*p < 0.05). The results shown are representative of three independent experiments. In all cases, cells were harvested 24 h post-transfection and the expression of fusion proteins was assessed by western blot using an anti-GFP antibody, which recognizes all fluorescent tagging proteins. β-Gal acted as a control for transfection efficiency and γ tubulin levels as loading control. Full-length blots are presented in the Supplementary figure S3

Equal amounts of protein extracts were separated by SDS-PAGE and transferred onto nitrocellulose membranes, as described previously [5]. Protein levels were ascertained by immunoblotting analysis using the appropriate primary antibodies, as indicated in the text. Blots were developed using SuperSignal West Pico Chemiluminescent Substrate reagent (Thermo Fisher Scientific, Rockford, IL, USA). Pictures were captured using Rx films (Fig. 1) or acquired with a GE Amersham Imager 600 equipment. Protein band intensities were quantified using the FIJI software [27]. In order to test statistical significance of protein variations one tailed t-test was performed with a significance level of *p* < 0.05.

### Fluorescence microscopy and immunofluorescence

HEK293 cells were grown on glass coverslips (350,000 cells/35 mm diameter dish), transfected for 24 h, and fixed using 4% paraformaldehyde in phosphate-buffered saline (PBS) for 30 min at room temperature.

Cell cultures overexpressing only fluorescent fusion proteins were directly mounted with SlowFade reagent (Molecular Probes, Thermo Fisher Scientific, USA). Cell cultures overexpressing HA-DLG1 were permeabilized with TritonX-100 0.1% in PBS after fixation, and incubated overnight with anti-HA (12CA5 clone, Sigma Aldrich, St. Louis, MO, USA). Cy3 conjugated anti-mouse IgG, was used as secondary antibody (Chemicon International, Temecula, CA, USA).

In all fluorescent microscopy experiments, despite the different spectral properties of the tags eventually used for protein detection, E6 expression was shown in green, E7 expression in blue and DLG1 expression in red. The Pearson correlation coefficient (PCC), used as co-localization parameter, was performed employing the Coloc2 plugin from FIJI software [27].

Fluorescence microscopy images were collected with a Carl Zeiss LSM880 confocal microscope following the sequential acquisition mode (Carl Zeiss, Germany). A 63x NA 1.4 plan apochromat oil immersion objective was employed.

.

### Immunoprecipitation

HEK293 cells co-transfected with HA-DLG1, seyfp-E618 and egfp-E718 expression plasmids were lysed with RIPA buffer (20 μM Tris pH = 7,4, 100 μM NaCl, 1% (v/v) Triton X-100, 0,5% (p/v) NaDC, 0.05% SDS) containing 1% Halt Protease inhibitor cocktail (Thermo Fisher Scientific Pierce, Rockford, IL, USA). Protein extracts were incubated overnight with anti-HA antibody. Afterwards, Protein A-Sepharose beads CL-4B (Sigma Aldrich, Saint Louis, MO, USA) were added to the extract and incubated in agitation for 3 h. The beads were subsequently separated by centrifugation at 15000 g, washed extensively with RIPA, and the bound immunocomplexes were eluted with SDS-sample buffer. The presence of HPV viral proteins in HA-DLG1 immunoprecipitates was ascertained by western blot using anti-GFP.

### RNA isolation, cDNA synthesis, and RT-qPCR

RNA purification was carried out using Trizol reagent according to the manufacturer’s protocol (Thermo Fisher Scientific, USA). Synthesis of cDNA was performed using 2 μg of RNA, 200 U MMuLV RevertAid reverse transcriptase (Thermo Fisher Scientific, Rockford, IL, USA) and oligo (dT) primers. RT-qPCR analysis was performed using 2 μl of cDNA and Eva Green qPCR Mezcla Real (Biodynamics, Buenos Aires, Argentina). The primers used for detection of DLG1 transcripts were: 5′-CAA GCA GCC TTA GCC CTA GTG TA-3′ (sense) and 5′-CAT GAA CCA ATT CTG GAC CTA TCA-3′ (antisense). For normalization, the transcripts’ levels of the reference gene succinate dehydrogenase (SDH) were quantified using SDH-F 5′ -GCA CAC CCT GTC CTT TGT-3′ (sense) and SDH-R 5′ -CAC AGT CAG CCT CGT TCA-3′ (antisense) oligonucleotides. The reaction conditions for *DLG1* and *SDH* detection were set at 95 °C for 5 min followed by 40 cycles of denaturation (95 °C for 15 s), annealing (58 °C for 15 s) and extension (72 °C for 20 s) with a single acquisition of fluorescence levels at the end of each extension step. Melting curve analysis was performed at the end of each qPCR reaction to ensure the amplification and detection of the correct PCR product. For RT-qPCR data analysis, the ΔΔCt relative quantification methods were used [28].

## Results

### DLG1 and E618 expression levels are highly dependent on their relative abundance

As expressed before, the relationship between high-risk HPV E6 and DLG1 may be complex, and the interaction between these proteins may not result, in all cases, in the degradation of the polarity protein, however, it could have differential consequences depending on the cellular context. Moreover, the levels and localization of these proteins change during the evolution of HPV-associated intraepithelial lesions [16, 29]. Hence, we aimed to investigate how variations in the abundance of one protein could affect the expression of the other one. We performed co-transfection experiments in HEK293 epithelial cells using different ratios of encoding vectors for E618 and DLG1, in order to obtain different relative amounts of these proteins. After 24 h, the cells were harvested and the protein levels were ascertained by western blot analysis. The results indicate that a high E618/DLG1 plasmid transfection ratio (Fig. 1a, left and middle panel) promotes a significant decrease in the levels of ectopic DLG1. However, this effect is no longer evident when the amount of transfecting vectors is equivalent (Fig. 1a, left panel). To fully corroborate this novel finding we quantified the intensity of DLG1 bands in this experimental condition from three independent experiments. As can be seen in Fig. 1a (right panel) the abundance of DLG1 did not significantly change, suggesting that a higher E6:DLG1 level ratio is required to target DLG1 for proteasome degradation. The same results were obtained when the experiment was performed in A549 epithelial cells, indicating that the effect of E618 over DLG1 expression levels is cell line independent (Fig S1). Interestingly, a low E618/ DLG1 ratio (a higher relative amount of DLG1) gives rise to increased levels of the viral protein (Fig. 1b). These data altogether strongly support the previously reported E618-mediated DLG1 degradation, and further demonstrate that this effect largely relies on high levels of E618 (Fig. 1, left panel). In addition, it is clearly shown that an increment in DLG1 levels positively affect E618 expression. Therefore, different outcomes for E618 and DLG1 expression can be observed depending on the experimental setup and the biological conditions, being useful to reveal different functional associations between these proteins.

### The co-expression of DLG1 and E618 mainly alters the subcellular localization of E618 in a PDZ-dependent manner

**Fig. 2:**
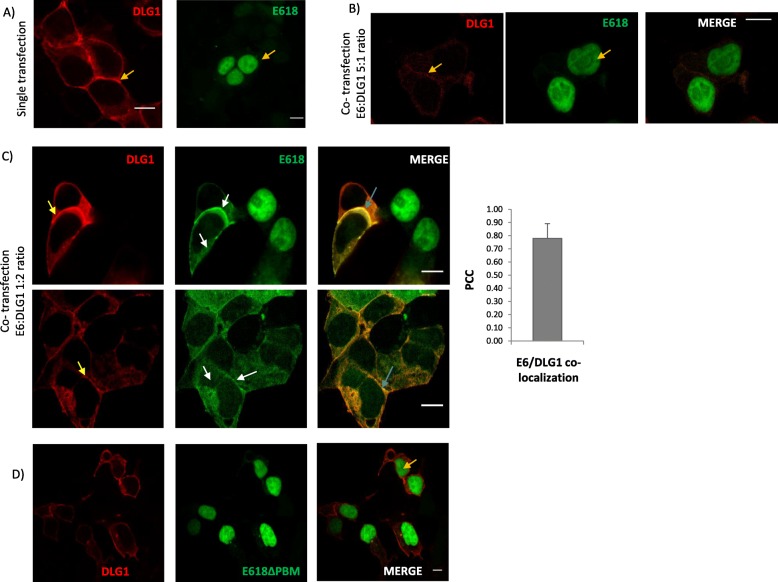
Analysis of the subcellular localization of E618 and DLG1 in co-expression experiments. **a** Subcellular distribution of E618 and DLG1 in epithelial cells. The encoding vectors for seyfp2-E618 or mTurq2-DLG1 were separately transfected into HEK293 cells and the localization of each fusion protein was analysed by confocal microscopy (yellow arrows). **b** DLG1 is barely expressed in the presence of high levels of E618. A 5:1 ratio of pseyfp2-E618:pmTurq2-DLG1 plasmids were used for transfection. Yellow arrows indicate the fusion protein localization. **c** Co-localization analysis of E618 with DLG1. A ratio of 1:2 of pseyfp2-E618:pmTurq2-DLG1 plasmids were used for transfection. White arrows indicate E618 redistribution. Light blue arrows indicate cellular regions where fusion proteins colocalize (see Merge image). PCC was calculated to evaluate seyfp2-E618/mTurq2-DLG1 colocalization, analysing a total of 90 micrographs from four independent experiments (right). **d** Analysis of E618 ΔPBM and mTurq2-DLG1 co-expression in epithelial cells. For the microscopy examination the same plasmid ratio as in (**c**) was used to co-transfect HEK293 cells. The yellow arrow shows the expression of seyfp2-E618ΔPBM in the nucleus. All scale bars represent 10 μm. The results are representative of at least 5 independent experiments

As a next step, we performed subcellular localization analyses of both proteins by confocal microscopy. HEK293 cells were selected since they proved to be useful in PDZ expression studies using fluorescence microscopy [30, 31]. Individual transfections with the encoding vectors for E618 and DLG1 fused to fluorescent proteins showed that the viral protein is mainly expressed in the nucleus while the PDZ protein is localized principally at the cell borders, as previously reported [32, 33] (Fig. 2a, yellow arrows). Interestingly, co-transfection experiments using the plasmid ratios described in Fig. 1 demonstrated changes in the expression of both proteins. In the E618 overexpression condition (Fig. 1a, left panel), DLG1 was barely detected by fluorescent microscopy, most probably due to the E6-mediated DLG1 degradation (Fig. 2b). In contrast, high levels of ectopic DLG1 promoted a striking relocalization of E618 out of the nucleus (Fig. 2c, white arrows, middle panel) whereas no significant changes in cell border expression of DLG1 were observed (Fig. 2c, yellow arrows, left panel). In addition, a confocal microscopy analysis of this condition revealed a clear co-localization pattern of DLG1 with E6 mainly at the cell contacts that was confirmed by the PCC examination (Fig. 2c, light blue arrow, right panel).

Next, we aimed to analyse whether this issue was dependent on the ability of E6 to interact with PDZ domains. For this, we generated a mutant version of E618 in which the E6 PBM sequence, ETQV, was deleted [5, 34] . This version, named E618ΔPBM, showed identical subcellular expression as the wild type E618, however it was unaffected by DLG1 overexpression (Fig. 2d, yellow arrow, right panel). Moreover, no evident co-localization of both proteins was observed.

Taken together, these data suggest that in these experimental conditions, E618-DLG1 generates a well-defined and specific co-localization pattern where the association of both proteins mainly affects the cell distribution of the viral protein, in a PDZ-dependent manner.

### The co-expression of HPV-18 E6 and E7 proteins promotes the subcellular redistribution of DLG1

As mentioned before, DLG1 deregulation has been observed in histological samples and organotypic raft cultures, which expressed not only E6 but also the HPV-E7 oncoprotein [16–18]. Therefore, we wanted to investigate in more detail the specific contribution of E7 to the observed changes in DLG1 expression.

**Fig. 3:**
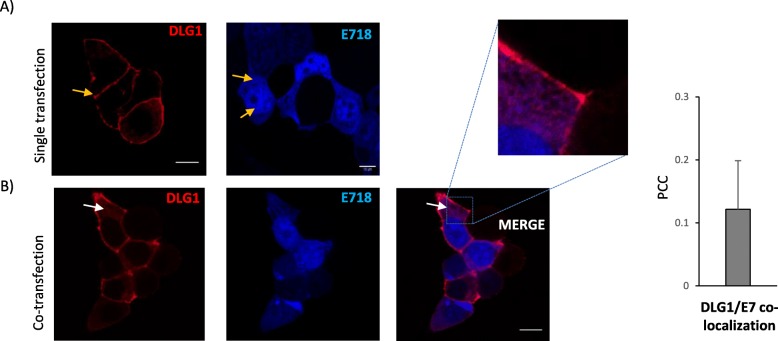
Analysis of DLG1 expression in the presence of E718. **a** Expression of mCherry-E718 in epithelial cells. HEK293 cells were transfected with pLPC-mCherry-E718 or pmTurq2-DLG1 vectors and analysed by confocal microscopy. The yellow arrows indicate the normal expression of mCherry-E718 (right) and mTurq2-DLG1 (left). **b** Simultaneous expression of E718 and DLG1 in epithelial cells. The white arrow indicates the delocalization of DLG1. PCC was calculated to evaluate mCherry-E718/mTurq2-DLG1 co-localization (right). The results are representative of at least 4 independent experiments. All scale bars: 10 μm

First, E718 and DLG1 expression vectors were separately transfected into HEK293 cells and the subcellular distribution of each protein was examined by confocal fluorescence microscopy. As can be observed, E718 (blue) was localized in the nucleus and the cytoplasm, as previously reported (Fig. 3a, yellow arrows) [35] whereas, DLG1 (red) was primarily located at the cell borders, as showed before (Fig. 3a, yellow arrow). Then, cells were co-transfected with the expression plasmids to evaluate the potential contribution of E718 to changes in DLG1 expression (Fig. 3b). A minor alteration in DLG1 subcellular localization, which presented a slightly diffused distribution pattern, was observed in the presence of E718 (compares Fig. 3a with Fig. 3b, yellow and white arrows, respectively).

**Fig. 4:**
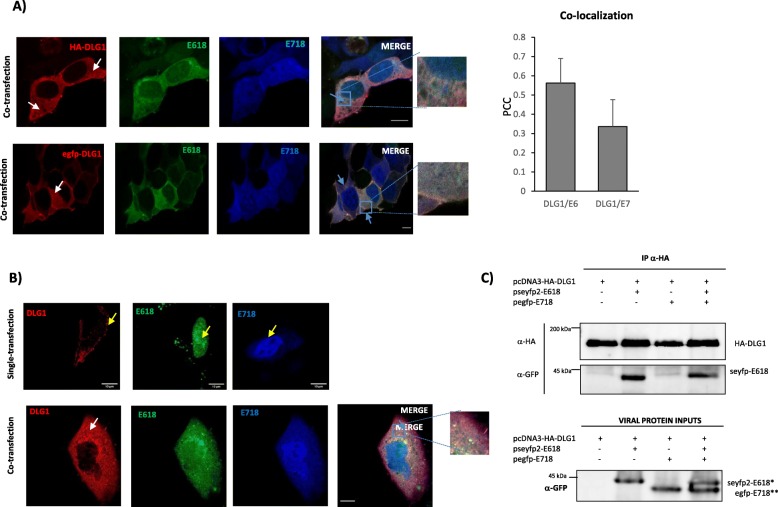
Analysis of DLG1 expression in the presence of both E618 and E718 oncoproteins. **a** Influence of E618 and E718 co-expression on DLG1 localization. The vectors for HA-DLG1 (red), mTurq2-E618 (green) and egfp-E718 (blue) expression were co-transfected in HEK293 cells. Cells were fixed, incubated overnight with an anti-HA antibody and counterstained with Cy3-tagged anti-mouse antibody (upper panel). HEK293 cells were transfected with expression vectors for fluorescence-tagged egfp-DLG1 (red), mTurq2-E6.18 (green) and cherry-E718 (blue) and analysed by confocal microscopy (bottom panel). White arrow indicates DLG1 redistribution. Light-blue arrow indicates DLG1 expression partially overlapped with E618 and E718 expression. Scale bars: 10 μm. The results are representative of at least 6 independent experiments and around 80 triple transfected cells were analysed. PCC was calculated to evaluate E718/DLG1 or E618/DLG1 co-localization (right). The results are representative of at least 6 independent experiments. **b** Influence of E618 and E718 co-expression on DLG1 localization in A549 cells. Upper panel: the encoding vectors for seyfp2-E618 or cherry-E718 or mTurq2-DLG1 were separately transfected into A549 cells and the localization of each fusion protein was analysed by confocal microscopy (yellow arrows). Bottom panel: A549 cells were transfected with expression vectors for fluorescence-tagged egfp-DLG1 (red), mTurq2-E618 (green) and cherry-E718 (blue) and analysed by confocal microscopy. White arrow indicates DLG1 redistribution (bottom panel). Light blue arrow indicates HA-DLG1 expression partially overlapped with E618 and E718 expression. Scale bars: 10 μm. **c** E718 does not co-immunoprecipitate with E618/DLG1 protein complexes. HEK293 cells were transfected with pcDNA3-HA-DLG1, pseyfp2-E618 and pegfp-E718 vectors. Equal amounts of protein extracts were immunoprecipitated with anti-HA antibody and bound to Protein A-Sepharose affinity beads. Immunoprecipitates, and a fraction of whole cell extracts (viral protein inputs), were resolved by SDS-PAGE. HA-DLG1 and viral proteins were probed with anti-HA and anti-GFP, respectively, by western blot. Full-lenght blots are presented in the Supplementary Figure S4. The results are representative of at least 3 independent experiments

Having corroborated the effects of E718, we next analysed DLG1 expression in the presence of both HPV oncoproteins, as performed for our previous analysis in raft cultures [18] and as naturally occurs in histological HPV-infected cervical samples [16, 18]. For this, we carried out triple transfection assays in HEK293 cells with vectors expressing E618, E718 and DLG1. Interestingly, cells co-expressing the three proteins exhibited a remarkable redistribution of DLG1 to the cytoplasmic region (Fig. 4a, white arrows). Furthermore, both viral proteins were also diffusely expressed in the cytoplasm and, from the analysis of fluorescence images, a partial overlapping of E618/DLG1 complexes with E718 was appreciated (Fig. 4a, see Merge, light-blue arrow). DLG1 redistribution was observed despite the vector or fusion tag used for DLG1 expression (Fig. 4a upper and bottom panel). Moreover, the same result, related to DLG1 redistribution from the cell borders, was obtained when the triple transfection experiment was performed using the A549 epithelial cells, indicating that the effect of viral proteins over DLG1 expression is independent of the epithelial cell line (Fig. 4b).

We therefore wanted to address whether this particular pattern of expression was associated with the formation of a tripartite complex involving HPV oncoproteins and DLG1. To this end, we performed co-immunoprecipitation (IP) assays in cells transfected with DLG1 together with E618 and/or E718 expression plasmids. As expected, E618 was detected in DLG1 immunoprecipitates either when the viral protein was co-transfected alone or in conjunction with E718. This result agrees with previous reports that demonstrated the PDZ-mediated interaction of DLG1 with E618 [5] (Fig. 4c). However, E718 did not co-immunoprecipitate with DLG1 alone or in the presence of E618 (Fig. 4c), indicating that in these experimental conditions E718 neither interacts with DLG1 nor with the E618-DLG1 complex, and ruling out a triple protein association.

### HPV E7 protein is involved in DLG1 protein stabilization

As shown above, in a context where the E618 oncoprotein is highly expressed (plasmid ratio 5/1, Fig. 1a), the expression levels of ectopic DLG1 decrease, thus probably reflecting the proteasomal degradation of DLG1 stimulated by E618 [5]. However, in order to further understand the changes in DLG1 expression in an HPV background, we wanted to investigate the effect of E718 expression in such conditions.

**Fig. 5:**
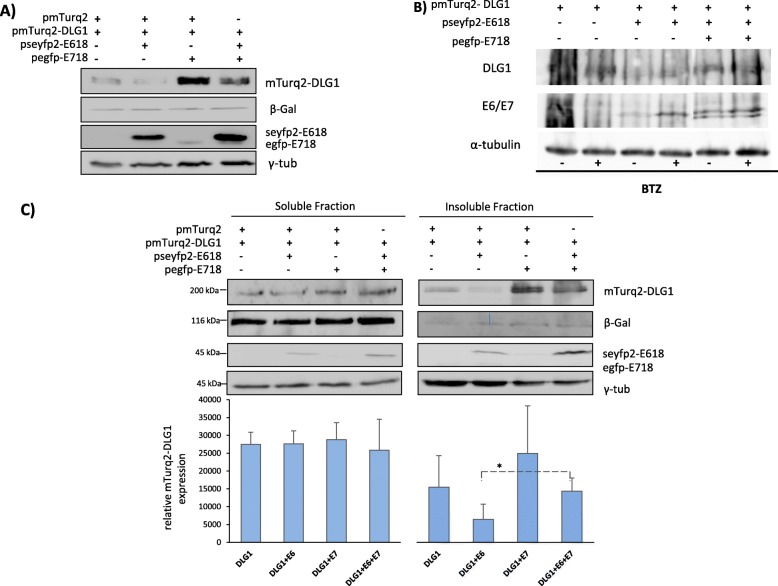
E718 restores DLG1 expression despite the presence of E618. **a** Analysis of total DLG1 expression levels in the presence of HPV-18 E6 and E7 proteins. HEK293 cells were transfected with 1 μg of pmTurq2-DLG1, 5 μg of pgw-HA-E618 and/or 5 μg of pegfp-E718 or pegfp-c1 empty vector (control). Fusion proteins were assessed by western blot using an anti-GFP antibody, which recognizes all fluorescent tagging proteins. γ tubulin levels were used as loading control. **b** HEK293 cells were transfected with 1 μg of pmTurq2-DLG1, 5 μg of pgw-HA-E618 and 5 μg of pegfp-E718 as indicated. The same amount of DNA was used for each transfection condition, using for balancing the pegfp-c1 empty vector. BTZ proteasome inhibitor was added to the cells 4 h before harvesting, as indicated (+). Fusion proteins was assessed by western blot using an anti-GFP antibody, which recognizes all fluorescent tagging proteins. α tubulin levels were used as loading control. **c** Changes in DLG1 levels in the soluble and insoluble fraction when E618 and E718 are co-expressed. HEK293 cells co-transfected with 1 μg of mTurq2-DLG1, 5 μg of seyfp2-E618, and/or 5 μg of pegfp-E718 were harvested, and the detergent insoluble and soluble fractions were obtained after centrifugation. Pegfp-c1 empty vector was used as control. Same amounts of protein extracts were resolved by SDS-PAGE and protein expression was ascertained by western blot using anti-GFP. Right, densitometry analysis of DLG1 levels normalized to γ-tubulin expression. Asterisks denote significant difference among conditions tested by one-tailed t-test (*p < 0.05). In **a** and **b**, the results shown are representative of three independent experiments. In all cases, cells were harvested after 24 hs post-transfection. β-Gal acted as a control for transfection efficiency in all assays. γ tubulin levels were used as loading control. Full-lenght blots are presented in the Supplementary Figure S5

In this case, as indicated in Fig. 5a, cells were again triple transfected with the E618, E718 and DLG1 expression plasmids. After 24 h, cells were harvested and the levels of DLG1 were ascertained by western blot. The results indicate that, as expected, the levels of DLG1 were greatly reduced in the presence of E618. Interestingly, in such condition DLG1 levels were rescued by the expression of E718. To get more insight about this we repeated the experiment but including BTZ proteasome inhibitors prior to protein extraction of the transfected cells. The results shown in Fig. 5b indicate that, the addition of the BTZ results in a partial restoration of DLG1 protein levels in E6 expressing cells as expected [5]. Interestingly, DLG1 levels in the presence of E618 were also rescued by the co-expression of E718, suggesting that E718 may contribute to an increase or stabilization of DLG1, mostly likely due to mechanisms that decrease its proteasomal degradation, however, other pathways may also be involved.

To extend our findings about DLG1 expression in the presence of both viral proteins, we analysed the subcellular region where the stabilization of DLG1 by E718 takes place. E618, E718 and DLG1 expression vectors were transfected into HEK293 cells and the resulting protein extracts were divided into detergent soluble and insoluble fractions. The abundance of ectopic DLG1 in each pool was determined by western blot. The results in Fig. 5c show that the presence of E618 induces a decrease in DLG1 levels mostly in the insoluble compartment, when compared to cells transfected with the empty vector. However, the co-expression of E718 restores DLG1 abundance in the same cellular insoluble fraction, whilst DLG1 expression is quite unaffected in the soluble cell portion (Fig. 5c).

To further confirm this finding, we also analysed the impact of E618 and E718 proteins on endogenous DLG1. Figure S2A shows that HPV proteins also modified the abundance and protein fraction distribution of endogenous DLG1, as for ectopic DLG1, even though to a lesser extent. Since several reports have linked E7 activities with transcription regulation of diverse genes, we wanted to evaluate whether the increase in DLG1 abundance mediated by E718 was due to transcriptional upregulation. Endogenous DLG1 mRNA levels were measured by RT-qPCR in the presence of the viral proteins, using HEK293 transfected cells. As shown in Fig. S2B, no significant variations in DLG1 transcript rates were found in the E718 and/or E618 expressing cells, compared to the control. Thus, the changes observed in DLG1 protein levels may not be related to transcription stimulation and they would be most likely connected to translational or posttranslational mechanisms, in agreement with results described above.

In general, the data of this section suggest that HPV-18 E6 and E7 may have opposing activities in regards to the regulation of DLG1 levels and may cooperatively contribute to its subcellular redistribution in the HPV context.

## Discussion

In this report, we present new insights into the effect of HPV proteins over the expression of the PDZ polarity regulator DLG1. Recently, we showed that changes in the levels and cell distribution of DLG1 in HPV-associated lesions may have an important role in tumour progression [17]. Remarkably, the loss of DLG1 expression at cell to cell contact (normal localization), together with an increase in DLG1 levels and unusual cytoplasmic distribution [15, 16], were also detected in biopsies derived from other tumours, like colon adenocarcinoma [16, 36] and breast cancer [37, 38]. Despite the importance of this abnormal DLG1 redistribution during cancer development, the mechanisms behind this remain elusive. Different pathways may be involved in diverse cancers, and in the case of HPV-associated carcinomas, both E618 and E718 viral oncoproteins may directly or indirectly facilitate such alterations, as we have recently shown in organotypic tissue cultures [18]. As a consequence, we initiated a series of studies in order to understand how the viral oncoproteins could affect DLG1 expression in an HPV scenario.

First, we evaluated using different cell lines the contribution of E618 protein since initial studies had demonstrated a PDZ-dependent interaction with DLG1. The main outcome for this interaction was shown to be the proteasomal degradation of DLG1 in cell culture models [5]. However, subsequent studies demonstrated that such degradation may be incomplete because significant levels of DLG1 were still detectable in HPV-associated cervical cancer cell lines [39]. Moreover, different analysis in organotypic raft cultures and intraepithelial cervical lesions showed that, DLG1 is still expressed despite the presence of the high-risk HPV E6 protein, however with some alteration in its cell distribution [15, 17, 18, 20]. Interestingly, a biophysical characterization of the E618-DLG1 complex highlighted the dynamic nature of such binding, allowing us to consider different consequences of such interaction according to the biological context [40]. In this report, we show that the relative abundance of E618 and DLG1 is a determinant factor for defining the outcome of such interaction, which allows us to hypothesize a reciprocal regulation between these proteins. This observation is relevant since both proteins are differentially expressed during the development of HPV-associated lesions. Interestingly, the overexpression of DLG1 increased E618 levels. In this sense, it was suggested a significant role for PDZ polarity proteins in the definition of E6 levels, especially during the HPV-16 lifecycle [41]. It is likely that the E6-PDZ interaction prevents the proteasomal degradation of E6, thereby favouring its stabilization and abundance during viral replication and the development of HPV-induced pathologies.

A remarkable fact is that, in the conditions described in Fig. 2, the co-expression of DLG1 and E618 promotes a striking delocalization of E618 from the nucleus to the cytoplasm and cell borders region, as well as a clear DLG1/E618 colocalization pattern. These changes depend on the presence of an intact E6 PBM, suggesting that the binding of these proteins is responsible for these observations. In line with this, we have previously demonstrated that the Human T cells leukemia virus type 1 Tax protein is relocalized from its nuclear localization to vesicle-like structures into the cytoplasm due to a PBM-PDZ dependent interaction with DLG1 [26]. Hence, it is possible that the interaction of these viral proteins with DLG1 induces their translocation into the cytoplasm, and represents a common strategy of viral pathogenesis either to block the anti-proliferative properties of DLG1 or to take advantage of specific scaffolding activities of DLG1 in these biological conditions.

Interestingly, the presence of both E618 and E718 oncoproteins produces a striking delocalization of DLG1 expression. This suggests that E718 protein, which is expressed together with E618 during the HPV-induced tumourigenesis, also participates in the deregulation of DLG1, a fact that is consistent with the concept that both proteins cooperate and are complementary in many viral activities. HPV E7 is a multifunctional protein that interacts with a high number of cell substrates, mainly key regulators of cell proliferation [2]. Therefore, it is possible to speculate that E7 may also contribute to deregulate polarity proteins involved in signal transduction pathways that regulate cell cycle progression. In this regard, DLG1 deregulation to the cytoplasmic region has been shown to be important for HPV-positive carcinoma cell, as cytoplasmic DLG1 pools bound to E6 favor the activation of the GTPase RhoG, which in turn facilitates the invasive potential of these cells [39]. Remarkably, this is in agreement with our findings that an accumulation of DLG1 in cells from HPV-associated intraepithelial lesions contributes to the progression of such lesions, indicating that DLG1 redistribution is important for cervical carcinogenesis [17]. It is worth noting that the subcellular localization of DLG1 changes during the different phases of the epithelial cell cycle, being mainly localized in the cytoplasm during the S phase [10]. Precisely, one of the most relevant functions of HPV E7 is precisely the stimulation of the S phase entry for the establishment of a proliferative cell state that allows viral replication [2]. Thus, it is not surprising that E718 expression facilitates the presence of DLG1 in the cytoplasmic region, an effect that is even more evident when the E618 protein is also expressed.

Even though triple transfection experiments showed some degree of fluorescence overlapping among E618, E718 and DLG1, it was not possible to detect a tripartite protein complex. In fact, only E618 co-immunoprecipitated with DLG1, as previously reported [5]. Hereafter, the effects of E718 over DLG1 may be due to indirect pathways, and not to a direct binding. In contrast, E7, through its multiple interacting partners, may modify important signal transduction mechanisms that promote DLG1 misexpression.

It was well established that the overexpression of E618 in transfection assays promotes the degradation of DLG1 in a PDZ- dependent manner [5]. However, no data are available regarding the effect of E718 presence in such condition. This is significantly relevant since the viral proteins are simultaneously expressed in a normal viral infection. We performed in vivo degradation assays and found that the presence of E718 rescued the E6-mediated DLG1 degradation, as the addition of proteasome inhibitors (Fig. 5b). This result suggests that E7 may increase DLG1 protein levels probably by contributing to its stabilization and/or preventing its degradation. In line with this, it was recently reported that HPV E7 interacts with different components of the ubiquitin-proteasome system including proteins that reverse the ubiquitination process, and, in this way, E7 might interfere with the degradation of certain cellular substrates [42]. It is also possible that E7 induced signal pathways that drive changes in DLG1 posttranslational modifications, rendering it more resistant to proteasomal degradation. In this sense, DLG1 is phosphorylated in several amino acid residues in response to diverse kinases and, interestingly, the non-phosphorylated forms of DLG1 are more resistant to E6-mediated degradation [43]. Thus, E7 expression could promote DLG1 dephosphorylation by regulating signalling mechanisms involved in the phospho-status of DLG1.

Cell extract fractionation experiments indicate that the E618-mediated degradation of DLG1 is more evident in the insoluble fraction. In the same way, when both viral proteins are expressed, the E7-mediated DLG1 stabilization is clear in the same insoluble fraction. It is likely that the accumulation of DLG1 in the cytoplasm region, which is appreciated by fluorescence microscopy, represents the pool of DLG1 protein in association with cell components, most likely with the cytoskeleton compartments where DLG1 is known to interact [43]. Remarkably, the E6-mediated degradation of DLG1 and the rescue of its levels in the presence of E7 were also observed for endogenous DLG1 protein. E7 was shown to positively and negatively alters the transcription of several host cell genes, by regulating factors involved in chromatin structure and DNA methylation; moreover, E7 can interfere with the activity of many transcription factors [44]. Hence, we examined whether DLG1 abundance was regulated transcriptionally by the expression of E7; however, as demonstrated by molecular techniques, no changes in DLG1 transcripts levels were observed. This supports the idea that translational or posttranslational mechanisms may be involved in the E7-mediated increase of DLG1 expression, as discussed above.

## Conclusions

In summary, this study constitutes a step forward in the knowledge of how DLG1 expression is deregulated during cervical cancer development and the involvement of the HPV oncoproteins in such alteration. Further research on this matter would provide a better comprehension of both HPV pathogenesis and the precise role of the differential expression of DLG1 in tumour progression.

## Supplementary information

**Additional file 1: Figure S1.** DLG1 and E618 protein expression levels in A549 cells. DLG1 levels decrease when the E618 oncoprotein is expressed at high levels. Different pseyfp2-E618/pmTurq2-DLG1 plasmid DNA ratios were transfected into A549 epithelial cells (5:1 and 1:1 μg). pcDNA3 was used as empty vector control. β-Gal acted as a control for transfection efficiency. Full-length blots are presented in the Supplementary figure S6. **Figure S2.** A) E618 and E718 proteins modify the abundance of endogenous DLG1. HEK293 cells were transfected with pcDNA3-E618 and/or pcDNA3-His-E718, or pcDNA3 empty vector (control). At 24 h post-transfection, total, soluble and insoluble protein extracts were prepared and endogenous DLG1 levels were analysed by western blot using an anti-DLG1 antibody. γ tubulin levels were determined as loading control. Full-length blots are presented in the Supplementary figure S7. B) DLG1 transcript levels remain unaltered despite E618 and/or E718 expression. HEK293 cells were transfected with pmTurq2-E618 and/or pLPC-mCherry-E7 vectors. Relative changes in total DLG1 mRNA level were quantified by RT-qPCR as described in Materials and methods. DLG1 mRNA levels of non-transfected HEK293 was arbitrarily considered to be 1 (control). DLG1 mRNA contents were normalised to SDH reference gene mRNA. Results represent the mean ± SE from three independent experiments.

## Data Availability

All data generated or analysed during this study are included in this article.

## Abbreviations

DLG1: Human Disc Large 1
egfp: enhanced green fluorescent protein
HA: Influenza virus Hemagglutinin epitope
HPVs: Human Papillomavirus
HSIL: High-Grade SIL
IP: Inmunoprecipitation LSIL: Low Grade SIL
mCherry: cherry fluorescence protein
mTurq2: enhanced version of cyan fluorescent protein
PBM: PDZ-Binding Motif
PCC: Pearson Correlation Coefficient
PDZ: PSD-95/DLG/ZO-1 domains
seyfp2: super enhanced yellow fluorescent protein
SIL: Squamous Intraepithelial precursor Lesions
