# Supplementary material for: HPV E6 and E7 oncoproteins cooperatively alter the expression of Disc Large 1 polarity protein in epithelial cells
Source: BMC Cancer. 2020 Apr 7;20:293. doi: 10.1186/s12885-020-06778-5 (PMC7137215; doi:10.1186/s12885-020-06778-5)
Supplement: Supplementary file 1 — Additional file 1: Figure S1. DLG1 and E618 protein expression levels in A549 cells. DLG1 levels decrease when the E618 oncoprotein is expressed at high levels. Different pseyfp2-E618/pmTurq2-DLG1 plasmid DNA ratios were transfected into A549 epithelial cells (5:1 and 1:1 μg). pcDNA3 was used as empty vector control. β-Gal acted as a control for transfection efficiency. Full-length blots are presented in the Supplementary figure S6. Figure S2. A) E618 and E718 proteins modify the abundance of endogenous DLG1. HEK293 cells were transfected with pcDNA3-E618 and/or pcDNA3-His-E718, or pcDNA3 empty vector (control). At 24 h post-transfection, total, soluble and insoluble protein extracts were prepared and endogenous DLG1 levels were analysed by western blot using an anti-DLG1 antibody. γ tubulin levels were determined as loading control. Full-length blots are presented in the Supplementary figure S7. B) DLG1 transcript levels remain unaltered despite E618 and/or E718 expression. HEK293 cells were transfected with pmTurq2-E618 and/or pLPC-mCherry-E7 vectors. Relative changes in total DLG1 mRNA level were quantified by RT-qPCR as described in Materials and methods. DLG1 mRNA levels of non-transfected HEK293 was arbitrarily considered to be 1 (control). DLG1 mRNA contents were normalised to SDH reference gene mRNA. Results represent the mean ± SE from three independent experiments. [file 12885_2020_6778_MOESM1_ESM.pptx]

## Slide 1
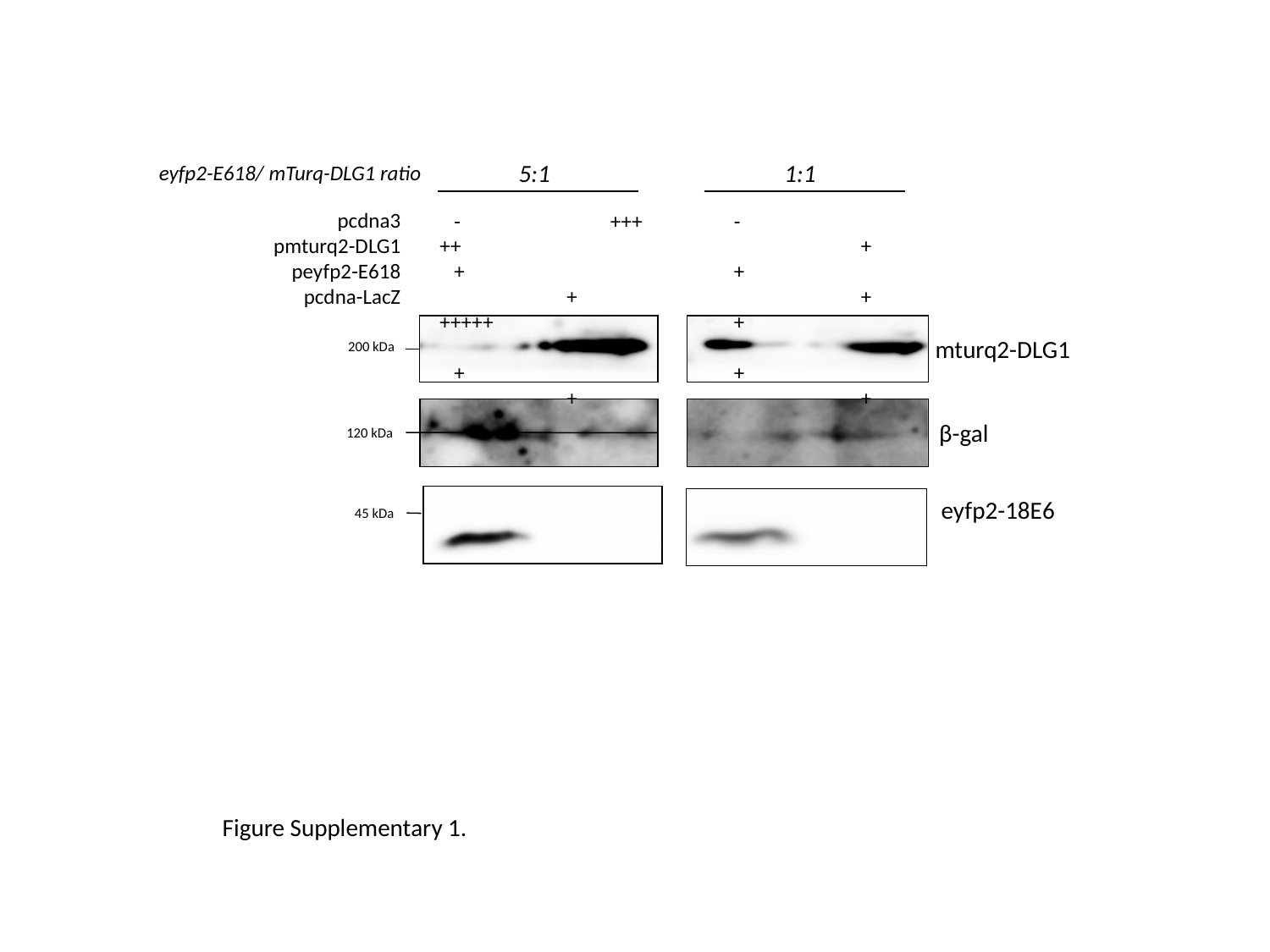

5:1
1:1
eyfp2-E618/ mTurq-DLG1 ratio
pcdna3
pmturq2-DLG1
peyfp2-E618
pcdna-LacZ
 -	 +++++
 +		+
+++++		-
 +		+
-	 	+
+		+
+		-
+		+
mturq2-DLG1
200 kDa
β-gal
120 kDa
eyfp2-18E6
45 kDa
Figure Supplementary 1.

## Slide 2
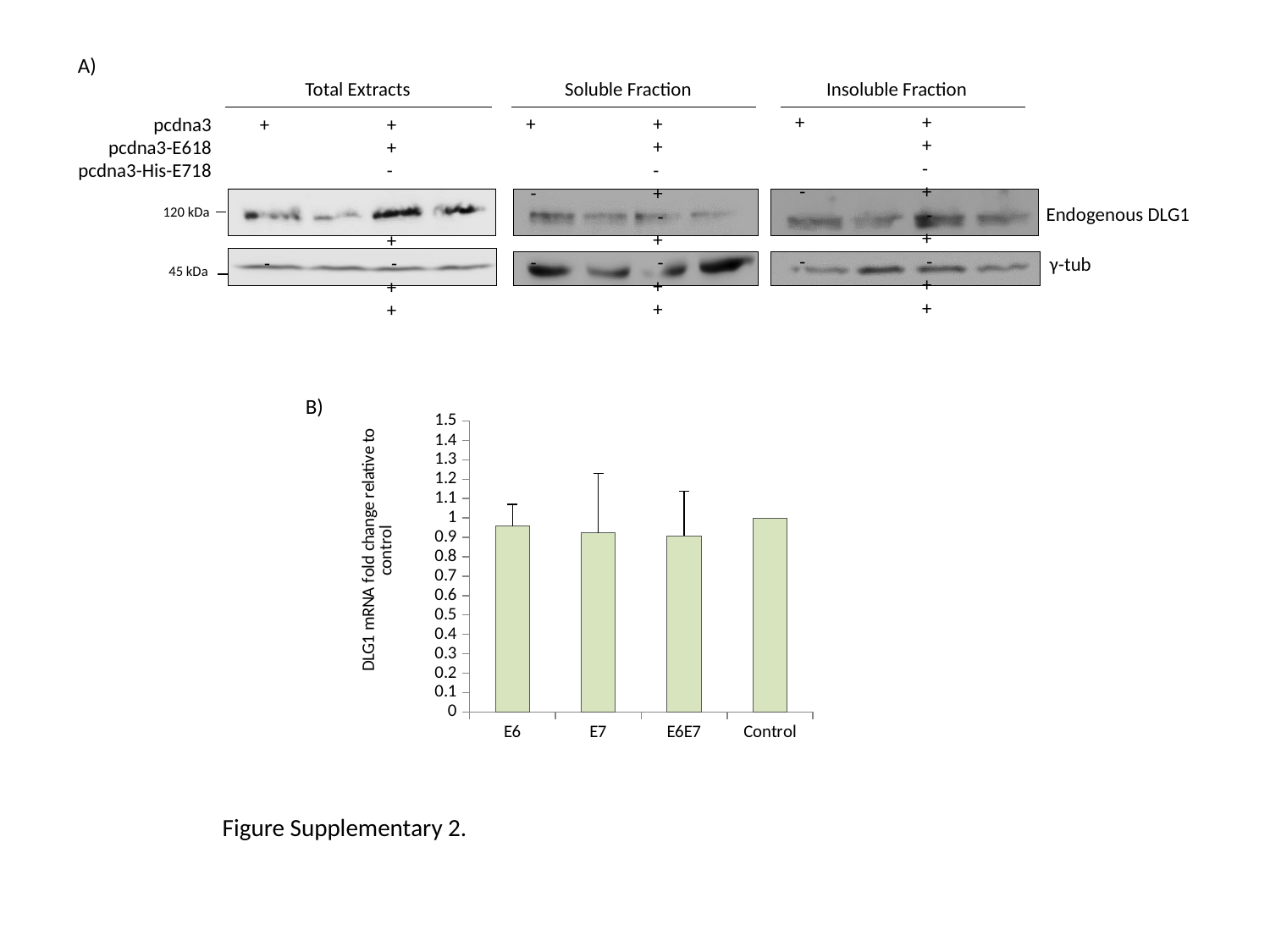

A)
Total Extracts
Soluble Fraction
Insoluble Fraction
+	+	+	-
 -	+	 -	+
 -	 -	+	+
+	+	+	-
 -	+	 -	+
 -	 -	+	+
pcdna3
pcdna3-E618
pcdna3-His-E718
+	+	+	-
 -	+	 -	+
 -	 -	+	+
Endogenous DLG1
120 kDa
γ-tub
45 kDa
### Chart
| Category | Cambio relativo |
|---|---|
| E6 | 0.9590000000000002 |
| E7 | 0.924 |
| E6E7 | 0.907 |
| Control | 1.0 |B)
Figure Supplementary 2.

## Slide 3
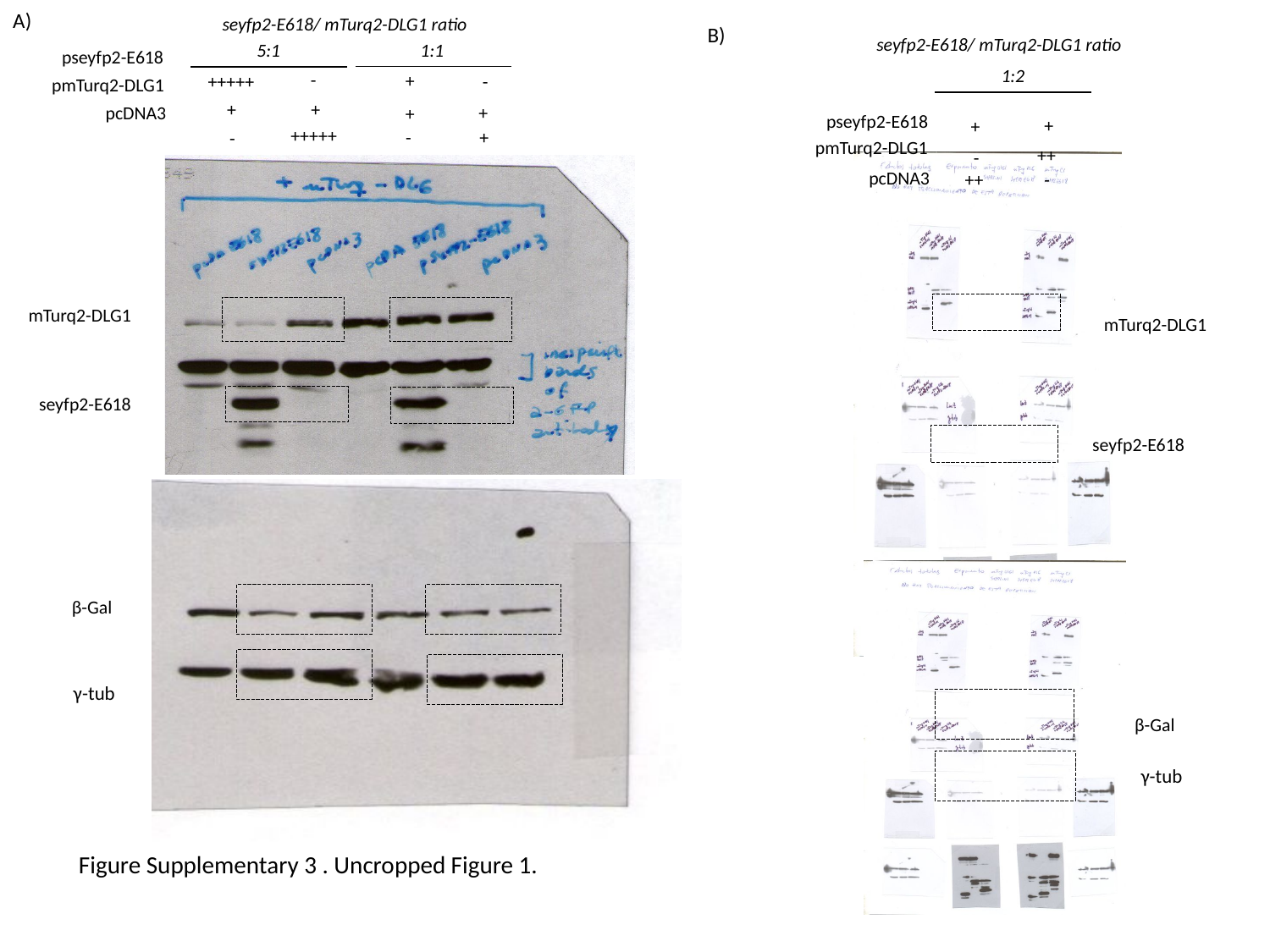

A)
seyfp2-E618/ mTurq2-DLG1 ratio
B)
seyfp2-E618/ mTurq2-DLG1 ratio
1:1
5:1
-
+
+++++
-
+
+
+
+
+++++
+
-
-
pseyfp2-E618
pmTurq2-DLG1
pcDNA3
1:2
pseyfp2-E618
+
+
pmTurq2-DLG1
++
-
pcDNA3
++
-
mTurq2-DLG1
mTurq2-DLG1
seyfp2-E618
seyfp2-E618
β-Gal
γ-tub
β-Gal
γ-tub
Figure Supplementary 3 . Uncropped Figure 1.

## Slide 4
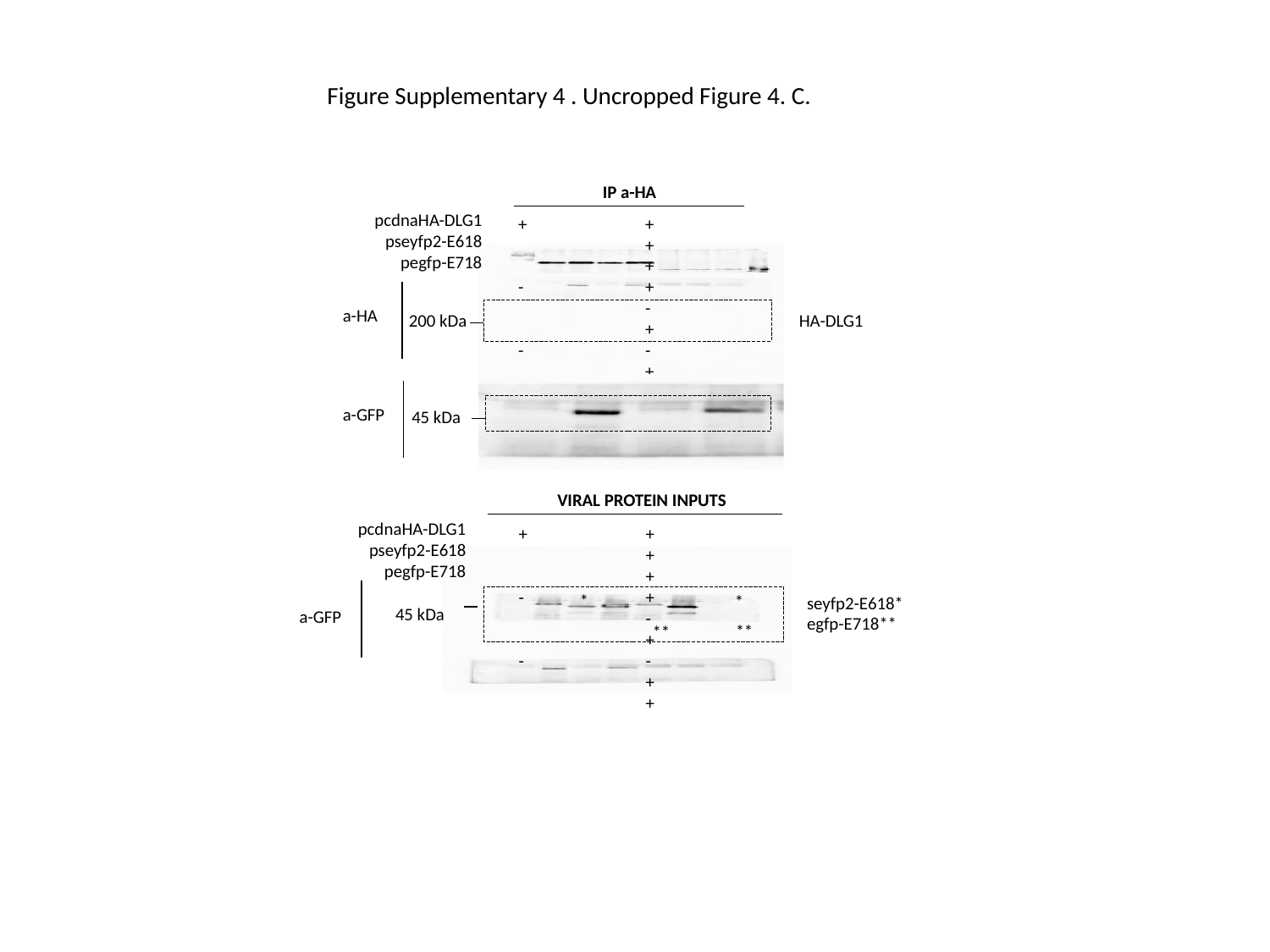

Figure Supplementary 4 . Uncropped Figure 4. C.
IP a-HA
pcdnaHA-DLG1
pseyfp2-E618
pegfp-E718
+	+	+	+
-	+	-	+
-	-	+	+
 a-HA
HA-DLG1
200 kDa
 a-GFP
45 kDa
VIRAL PROTEIN INPUTS
pcdnaHA-DLG1
pseyfp2-E618
pegfp-E718
+	+	+	+
-	+	-	+
-	-	+	+
*
*
seyfp2-E618*
egfp-E718**
45 kDa
 a-GFP
**
**

## Slide 5
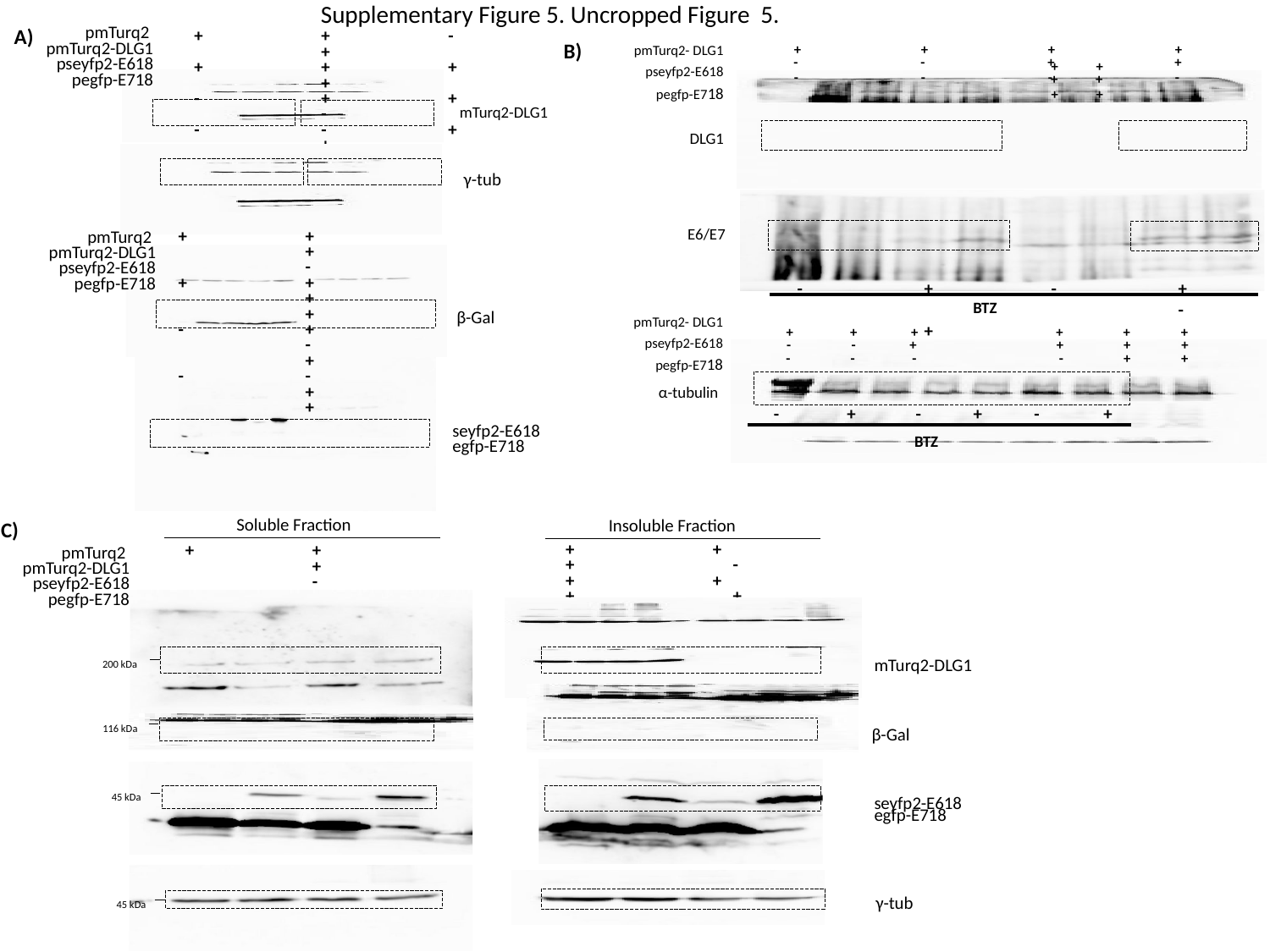

Supplementary Figure 5. Uncropped Figure 5.
A)
pmTurq2
pmTurq2-DLG1
pseyfp2-E618
pegfp-E718
+	+	-	+
+	+	+	+
-	+	+	-
-	-	+	+
B)
+	+	+	+ 		 + +
pmTurq2- DLG1
pseyfp2-E618
pegfp-E718
-	-	+	+ 		 + +
-	-	-	- 		 + +
DLG1
E6/E7
-	+	-	+			-	+
 BTZ
pmTurq2- DLG1
pseyfp2-E618
pegfp-E718
+ + +	 + + +
 - - +	 + + +
- - -	 - + +
- + - + - +
BTZ
α-tubulin
mTurq2-DLG1
γ-tub
+	+	+	-
+	+	+	+
-	+	-	+
-	-	+	+
pmTurq2
pmTurq2-DLG1
pseyfp2-E618
pegfp-E718
β-Gal
seyfp2-E618
egfp-E718
Soluble Fraction
Insoluble Fraction
+	 +	 +	 -
+	 +	 +	 +
-	 + - +
-	-	+	+
+	+	+	-
+	+	+	+
-	+	-	+
-	-	+	+
pmTurq2
pmTurq2-DLG1
pseyfp2-E618
pegfp-E718
200 kDa
mTurq2-DLG1
116 kDa
β-Gal
45 kDa
seyfp2-E618
egfp-E718
45 kDa
γ-tub
C)

## Slide 6
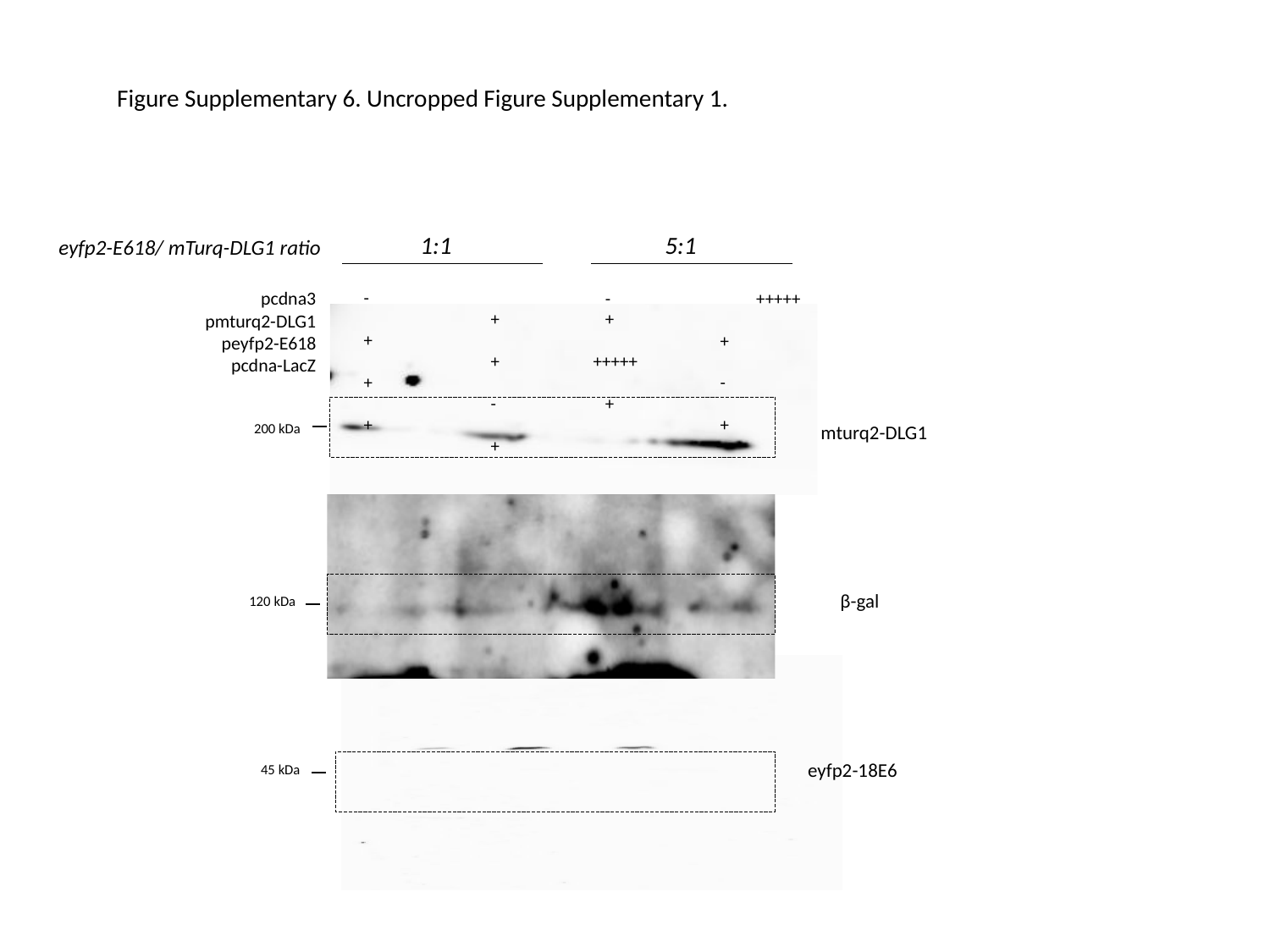

Figure Supplementary 6. Uncropped Figure Supplementary 1.
5:1
1:1
eyfp2-E618/ mTurq-DLG1 ratio
-	 	+
+		+
+		-
+		+
 -	 +++++
 +		+
+++++		-
 +		+
pcdna3
pmturq2-DLG1
peyfp2-E618
pcdna-LacZ
200 kDa
mturq2-DLG1
β-gal
120 kDa
eyfp2-18E6
45 kDa

## Slide 7
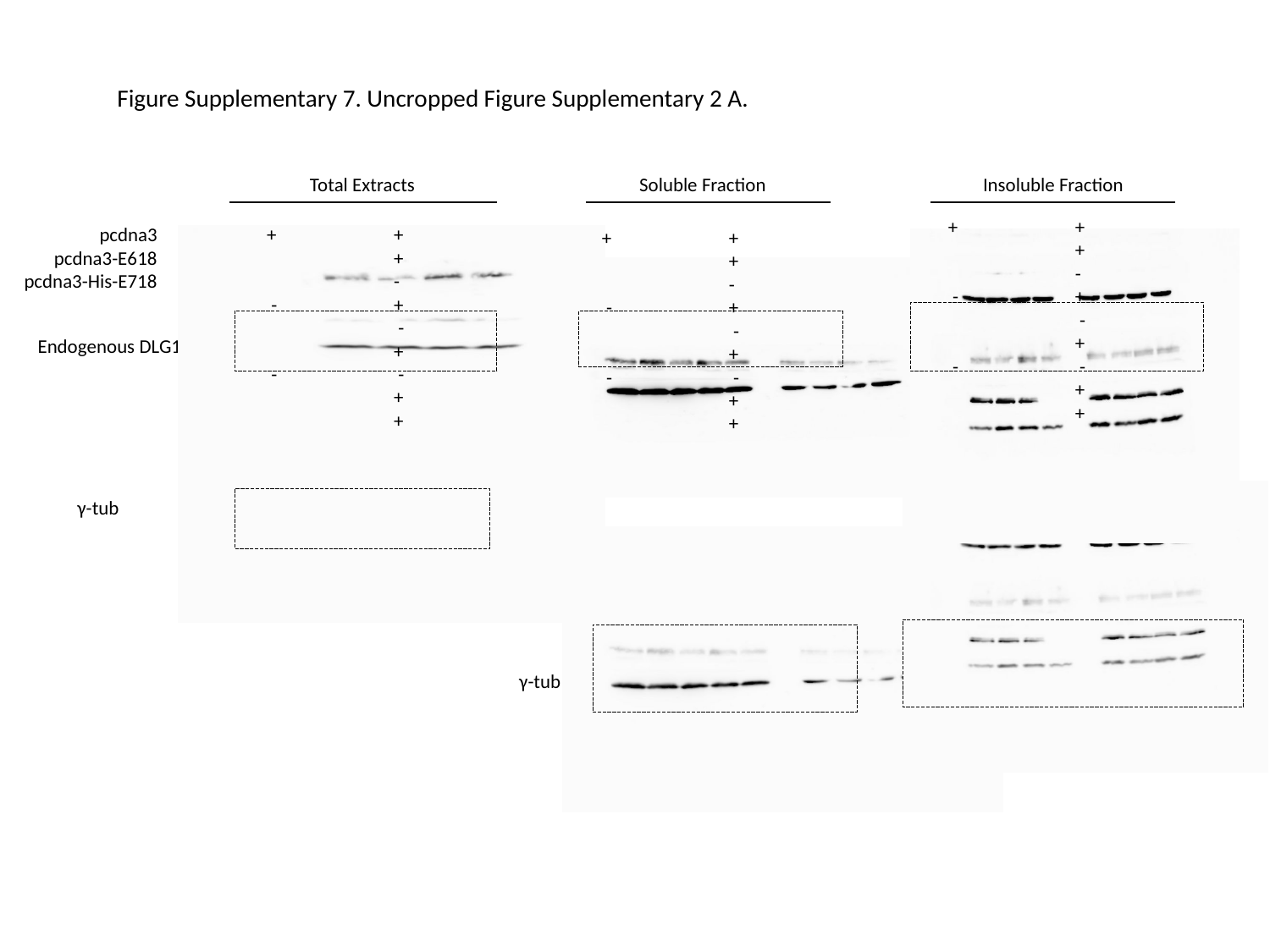

Figure Supplementary 7. Uncropped Figure Supplementary 2 A.
Total Extracts
+	+	+	-
 -	+	 -	+
 -	 -	+	+
pcdna3
pcdna3-E618
pcdna3-His-E718
Soluble Fraction
Insoluble Fraction
+	+	+	-
 -	+	 -	+
 -	 -	+	+
+	+	+	-
 -	+	 -	+
 -	 -	+	+
Endogenous DLG1
γ-tub
γ-tub
